# Self-Assembly of Linear Amphiphilic Pentablock Terpolymer PAA*_x_*-PS_48_-PEO_46_-PS_48_-PAA*_x_*in Dilute Aqueous Solution

**DOI:** 10.3390/polym12102183

**Published:** 2020-09-24

**Authors:** Jia Gao, Kun An, Chao Lv, Jingjing Nie, Junting Xu, Binyang Du

**Affiliations:** 1 MOE Key Laboratory of Macromolecular Synthesis and Functionalization, Department of Polymer Science & Engineering, Zhejiang University, Hangzhou 310027, China; 11629010@zju.edu.cn (J.G.); 21929006@zju.edu.cn (K.A.); 21629025@zju.edu.cn (C.L.); xujt@zju.edu.cn (J.X.); 2 Department of Chemistry, Zhejiang University, Hangzhou 310027, China; niejj@zju.edu.cn

**Keywords:** pentablock terpolymers, self-assembly, porous vesicles, double nanotubes

## Abstract

A series of linear amphiphilic pentablock terpolymer PAA*_x_*-*b*-PS_48_-*b*-PEO_46_-*b*-PS_48_-*b*-PAA*_x_* (A*_x_*S_48_O_46_S_48_A*_x_*) with various lengths *x* of the PAA block (*x* = 15, 40, 60, and 90) were synthesized via a two-step atom transfer radical polymerization (ATRP) using Br-poly(ethylene oxide)-Br (Br-PEO_46_-Br) as the macroinitiator, styrene (St) as the first monomer, and *t*ert-butyl acrylate (*t*BA) as the second monomer, followed with the hydrolysis of P*t*BA blocks. The A*_x_*S_48_O_46_S_48_A*_x_* pentablock terpolymers formed micelles in dilute aqueous solution, of which the morphologies were dependent on the length *x* of the PAA block. Cryogenic transmission electron microscopy (cryo-TEM), dynamic light scattering (DLS), and zeta potential measurement were employed to investigate the morphologies, chain structures, size, and size distribution of the obtained micelles. The morphology of A*_x_*S_48_O_46_S_48_A*_x_* micelles changed from spherical vesicles with ordered porous membranes to long double nanotubes, then to long nanotubes with inner modulated nanotubes or short nanotubes, and finally, to spherical micelles or large compound vesicles with spherical micelles inside when *x* increased from 15 to 90. The hydrophobic PS blocks formed the walls of vesicles and nanotubes as well as the core of spherical micelles. The hydrophilic PEO and PAA block chains were located on the surfaces of vesicle membranes, nanotubes, and spherical micelles. The PAA block chains were partially ionized, leading to the negative zeta potential of A*_x_*S_48_O_46_S_48_A*_x_* micelles in dilute aqueous solutions.

## 1. Introduction

Amphiphilic block copolymers, with hydrophilic and hydrophobic segments connected by covalent bonds, can self-assemble into micelles with various morphologies and structures in selective solvents [1,2,3,4]. Because of the unique physical and chemical properties, block copolymer micelles have been used in many technical fields, including drug delivery [5,6], templates [7,8,9], hydrogels [10,11], and membranes [12,13,14]. In past decades, the self-assembly of block copolymers in selective solvents has been extensively studied experimentally [15,16] and theoretically [17,18,19,20]. The morphologies and structures of block copolymer micelles are strongly dependent on the monomer type, composition, and molecular weight of block copolymers as well as the use of selective solvents. Spherical and rod-like micelles, vesicles, large compound micelles, micelles with octopi, jellyfish, and bamboo cage shapes as well as spindle worm-like micelles have been reported for AB diblock copolymer and ABC triblock terpolymers in various selective solvents like aqueous solution, DMF, methanol-water mixture, and THF-water mixture [21,22,23,24,25].

Recently, the self-assembly of linear ABCBA pentablock terpolymers in selective solvents has attracted increasing interest. The ABCBA pentablock terpolymers were thought to serve as the simplest model system of multiblock copolymers, which provide more controllable structure parameters like number, sequence, and composition of the blocks. The investigation of self-assembly multiblock copolymers in solutions will shed light on the understanding of solution properties of biomacromolecules [26,27]. How to systematically manipulate chain architectures, assembly behavior, and microstructures of multiblock copolymers in solutions has been becoming an active area [28,29,30]. For instance, Zhang et al. [28] synthesized poly(2-dimethylaminoethyl methacrylate)-*block*-poly(2,2,2-trifluoroethylmethacrylate)-*block*-poly(e-caprolactone)-*block*-poly(2,2,2-trifluoroethylmethacrylate)-*block*-poly(2-dimethylaminoethyl methacrylate) (PDMAEMA-*b*-PTFEMA-*b*-PCL-*b*-PTFEMA-*b*-PDMAEMA) pentablock terpolymers via a two-step atom transfer radical polymerization (ATRP) and obtained spherical micelles in aqueous solution with different pH values. They found that the mean diameter of micelles increased with the decrease in pH value. Mallapragada et al. [31,32] reported the syntheses of a series of pentablock terpolymers by using modified poly(ethylene oxide)_100_-poly(propylene oxide)_65_-poly(ethylene oxide)_100_ (PEO_100_-PPO_65_-PEO_100_ or F127) as the macroinitiator and 2-(diethylamino)ethyl methacrylate (DEAEMA), DMAEMA, 2-diisopropylaminoethyl methacrylate (DiPAEMA), or (*tert*-butylamino)ethyl methacrylate (*t*BAEMA) as the monomer and their formation of thermo- and pH-responsive micelles in dilute aqueous solutions. Thunemann et al. [29] reported the formation of two-compartment cylindrical micelles for poly(ethylene oxide)-*block*-poly(γ-benzyl *L*-glutamate)-*block*-poly(perfluoro ether)-*block*-poly(γ-benzyl *L*-glutamate)-*block*-poly(ethylene oxide) (PEO-*b*-PBLG-*b*-PFPE-*b*-PBLG-*b*-PEO) pentablock terpolymers in aqueous solutions. The formation of thermosensitive spherical micelles of poly(*N*-isopropylacrylamide)-*block*-poly(ethylene oxide)-*block*-poly(propylene oxide)-*block*-poly(ethylene oxide)-*block*-poly(*N*-isopropylacrylamide) (PNIPAM-*b*-PEO-*b*-PPO-*b*-PEO-*b*-PNIPAM) pentablock terpolymers in aqueous solutions have been reported by Du et al. [33,34] and Parekh et al. [30]. Lv et al. [35] reported that poly(*N*-isopropylacrylamide)_x_-*b*-poly(*t*ert-butyl acrylate)_90_-*b*-poly(propylene oxide)_36_-*b*-poly(*t*ert-butyl acrylate)_90_-*b*-poly(*N*-isopropylacrylamide)_x_ (PNIPAM*_x_*-*b*-P*t*BA_90_-*b*-PPO_36_-*b*-P*t*BA_90_-*b*-PNIPAM*_x_*, *x* = 111, 107, 95, 58, 33, and 26) formed core-corona spherical micelles with a homogeneous hydrophobic core, rod-like micelles, core-shell-corona spherical micelles with clear or blurry boundaries, and large compound spherical micelles with embedded hydrophilic chains in the hydrophobic region, respectively, when the length *x* of PNIPAM blocks decreased from 111 to 26.

However, up to now, most ABCBA pentablock terpolymer systems reported were mainly designed to be hydrophilic–hydrophobic–hydrophilic or hydrophobic–hydrophilic–hydrophobic, where A and B blocks or B and C blocks were both soluble or insoluble in aqueous solution at room temperature. The study of ABCBA pentablock terpolymers with alternated hydrophilicity, where A and C blocks are water soluble with water-insoluble B block or vice versa, is still rare. Furthermore, the most reported self-assembly of ABCBA pentablock terpolymers mainly focused on the stimuli-responsiveness of micelles in aqueous solution instead of finding new micelle morphologies. Therefore, we design a new ABCBA pentablock terpolymer with hydrophobic PS as B block and hydrophilic PAA and PEO as A and C blocks in the present work. Four linear ABCBA-type amphiphilic pentablock terpolymers, poly(acrylic acid)*_x_*-*b*-poly(styrene)_48_-*b*-poly(ethylene oxide)_46_-*b*-poly(styrene)_48_-*b*-poly(acrylic acid)*_x_* (PAA*_x_*-*b*-PS_48_-*b*-PEO_46_-*b*-PS_48_-*b*-PAA*_x_* or A*_x_*S_48_O_46_S_48_A*_x_*) with various lengths *x* of PAA block (*x* = 15, 40, 60, and 90) were synthesized by a two-step ATRP process followed with hydrolysis treatment. A*_x_*S_48_O_46_S_48_A*_x_* formed micelles in dilute aqueous solutions, which were investigated by cryogenic transmission electron microscopy (cryo-TEM), dynamic light scattering (DLS), and Zeta potential measurement. Interestingly, new micelle morphologies, namely long double nanotubes with different inner nanotube structures, were observed for A_40_S_48_O_46_S_48_A_40_ and A_60_S_48_O_46_S_48_A_60_. The effects of PAA block length *x* on the morphologies and chain structures of A*_x_*S_48_O_46_S_48_A*_x_* micelles in dilute aqueous solutions were discussed.

## 2. Materials and Methods

### 2.1. Materials

*Tert*-Butyl acrylate (*t*BA, 99%), 2-bromoisobutyryl bromide (BIBB, 98%), *N,N,N**′**,N**″**,N**″***-pentamethyldiethylenetriamine (PMDETA, 98%), copper(I) bromide (CuBr, 98%), trifluoroacetic acid (TFA, 99.5%), 4-dimethylaminopyridine (DMAP, 98%), and triethylamine (TEA, ultra-dry) were purchased from J&K Chemical Ltd., Shanghai, China. Styrene (St, 99%) and poly(ethylene oxide) (HO-PEO_46_-OH, *M_n_* = 2050, *M_w_/M_n_* = 1.06) were purchased from Sinopharm Chemical Reagent Co., Ltd., Shanghai, China, and Sigma-Aldrich Chemical Ltd., St. Louis, MO, USA, respectively. St and *t*BA were purified by passing through alkaline alumina column (200–300 mesh), respectively. CuBr was purified by sequential washing with glacial acetic acid, absolute ethanol, and anhydrous ether, followed by drying in a vacuum overnight. The purified CuBr was sealed in a brown bottle for further usage. Other chemicals and solvents with analytical grade were used as received.

### 2.2. Synthesis of Br-PEO_46_-Br Macroinitiator

Macroinitiator, Br-PEO_46_-Br, was synthesized by reacting HO-PEO_46_-OH and BIBB, with DMAP and TEA as catalysts in anhydrous dichloromethane (DCM). Briefly, 20.5 g HO-PEO_46_-OH, 0.2 g DMAP, and 8.5 mL TEA were completely dissolved in 100 mL DCM under nitrogen atmosphere in an ice/water bath. Then, 10 mL BIBB DCM solutions (4 mol/L) were dropwise added into the mixed solution. After 2 h, the ice/water bath was taken away and the reaction continued for 24 h at room temperature. Afterward, the mixture was filtered and washed with saturated NaHCO_3_ solution and deionized water. The mixture was further dried with anhydrous Na_2_SO_4_ powders overnight. The Na_2_SO_4_ powders were filtered and the residue dark-brown solution was concentrated and then, precipitated in cold *n*-hexane. The obtained light-yellow powders were dried at 40 °C under vacuum overnight to give the macroinitiator, Br-PEO_46_-Br. A ^1^H NMR spectrum of Br-PEO_46_-Br macroinitiator indicated the bromination extent of PEO_46_ was 100%, as shown in Figure S1.

### 2.3. Synthesis of PtBA_x_-PS_48_-PEO_46_-PS_48_-PtBA_x_ Pentablock Terpolymers

The pentablock terpolymers P*t*BA*_x_*-PS_48_-PEO_46_-PS_48_-P*t*BA*_x_* with various P*t*BA block lengths *x* and fixed PS block length of 48 were synthesized via a two-step ATRP process using Br-PEO_46_-Br as the macroinitiator, St as the first monomer, and *t*BA as the second monomer. Firstly, 0.43 mmol Br-PEO_46_-Br, 262 mmol St, and 3.4 mmol PMDETA were completely mixed in a 50 mL Schlenk tube under nitrogen atmosphere. The mixture was degassed via freezing–pumping–thawing process three times. Then, 1.7 mmol CuBr was added and the Schlenk tube was sealed and then immersed into an oil bath with reset temperature of 90 °C. After 1 h, the reaction was quenched by putting the Schlenk tube in liquid nitrogen. The reaction mixture was passed through a neutral alumina column (100–200 mesh) by using DCM as the eluent. The purified mixture was concentrated and then precipitated in cold n-hexane. The resultant white precipitates were dried overnight at 40 °C under vacuum to give Br-PS_48_-PEO_46_-PS_48_-Br triblock copolymer.

The obtained Br-PS_48_-PEO_46_-PS_48_-Br triblock copolymer was used as the macroinitiator to further polymerize the second monomer, *t*BA, via a similar ATRP procedure. Note that the ATRP of *t*BA was carried out at 75 °C for 1 h. Four pentablock terpolymers, namely P*t*BA_15_-PS_48_-PEO_46_-PS_48_-P*t*BA_15_, P*t*BA_40_-PS_48_-PEO_46_-PS_48_-P*t*BA_40_, P*t*BA_60_-PS_48_-PEO_46_-PS_48_-P*t*BA_60_, and P*t*BA_90_-PS_48_-PEO_46_-PS_48_-P*t*BA_90_, were synthesized.

### 2.4. Preparation of PAA_x_-PS_48_-PEO_46_-PS_48_-PAA_x_ Pentablock Terpolymers

The PAA*_x_*-PS_48_-PEO_46_-PS_48_-PAA*_x_* pentablock terpolymers were prepared from the corresponding P*t*BA*_x_*-PS_48_-PEO_46_-PS_48_-P*t*BA*_x_* pentablock terpolymers via the hydrolysis of P*t*BA blocks. Briefly, given amounts of P*t*BA*_x_*-PS_48_-PEO_46_-PS_48_-P*t*BA*_x_* and TFA were dissolved in 2 mL DCM and stirred at room temperature for 24 h. The P*t*BA blocks were completely hydrolyzed to give PAA blocks. The hydrolysis products were precipitated in cold n-hexane and the precipitates were dried under vacuum overnight to give PAA*_x_*-PS_48_-PEO_46_-PS_48_-PAA*_x_* pentablock terpolymers.

The P*t*BA*_x_*-PS_48_-PEO_46_-PS_48_-P*t*BA*_x_* and PAA*_x_*-PS_48_-PEO_46_-PS_48_-PAA*_x_* pentablock terpolymers were then coded as T*_x_*S_48_O_46_S_48_T*_x_* and A*_x_*S_48_O_46_S_48_A*_x_*, respectively. The capital letters O, S, A, and T were used to represent the PEO, PS, PAA, and P*t*BA blocks, respectively.

### 2.5. Preparation of A_x_S_48_O_46_S_48_A_x_ Micelles in Dilute Aqueous Solutions

Certain amounts of A*_x_*S_48_O_46_S_48_A*_x_* pentablock terpolymers were completely dissolved in tetrahydrofuran (THF) to give a concentration of 1 mg/mL. Deionized water was then dropped into the A*_x_*S_48_O_46_S_48_A*_x_* THF solutions at a constant rate (ca. 1 drop per 10 s) under vigorous stirring until the water content reached 35 wt%. Afterward, the mixing solutions were transferred into a dialysis tube (molecular weight cutoff of 3500), which was then dialyzed against deionized water for 3 days. The final micelle solutions were transferred into a clean volumetric glass flask and diluted with deionized water to give a final concentration of 0.3 mg/mL. The pH values of micelle aqueous solutions were measured to be 5.23, 5.25, 5.20, and 5.12 for A_15_S_48_O_46_S_48_A_15_, A_40_S_48_O_46_S_48_A_40_, A_60_S_48_O_46_S_48_A_60_, and A_90_S_48_O_46_S_48_A_90_, respectively, by a pH meter (FE28, METTLER TOLEDO, Zurich, Switzerland).

### 2.6. Measurements

Gel permeation chromatograph (GPC, Waters 1515 system, Waters Corp., Milford, MA, USA) was used to measure the dispersities (*M_w_/M_n_*s) of T*_x_*S_48_O_46_S_48_T*_x_* pentablock terpolymers with polystyrene as the calibration standard and THF as the eluent (1.0 mL/min) at 40 °C. Number-average molecular weights (*M_n_*s) of T*_x_*S_48_O_46_S_48_T*_x_* and A*_x_*S_48_O_46_S_48_A*_x_* were determined from their ^1^H NMR spectra, which were recorded on a Bruker DMX-400 MHz instrument (Bruker Corp., Karlsruhe, Germany).

Dynamic light scattering (DLS) measurements of A*_x_*S_48_O_46_S_48_A*_x_* micelle solutions were performed on a Brookhaven BI-200SM Instrument (Brookhaven Instruments Corp., Holtsville, NY, USA). with a laser wavelength of 657 nm at 25 °C. The scattering angle was fixed at 90°. The Zeta potentials ξ of A*_x_*S_48_O_46_S_48_A*_x_* micelles were also measured by electrophoretic light scattering (ELS) using the same BI-200SM Instrument.

The morphologies of A*_x_*S_48_O_46_S_48_A*_x_* micelles were observed by cryogenic transmission electron microscopy (cryo-TEM) on a Talos F200C cryogenic electron microscopy (FEI Company, Hillsboro, Oregon) at an acceleration voltage of 200 kV. The micelle solutions were dropped onto bare copper grids, which were immediately submerged into liquid nitrogen for flash freezing. The cryo-grids were then mounted on a cryogenic sample holder. Images were recorded on a Ceta 4 K × 4 K camera with a resolution ratio ≤0.3 nm.

## 3. Results and Discussion

### 3.1. Synthesis and Characterization of A_x_S_48_O_46_S_48_A_x_ Pentablock Terpolymers

**Scheme 1:**
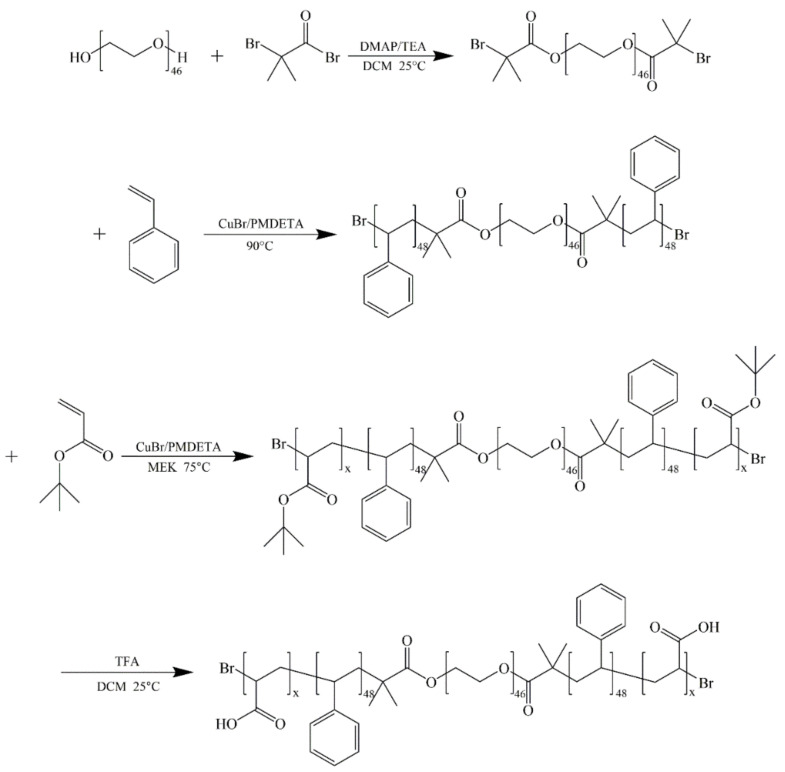
Synthesis route of A*_x_*S_48_O_46_S_48_A*_x_* pentablock terpolymers.

**Figure 1:**
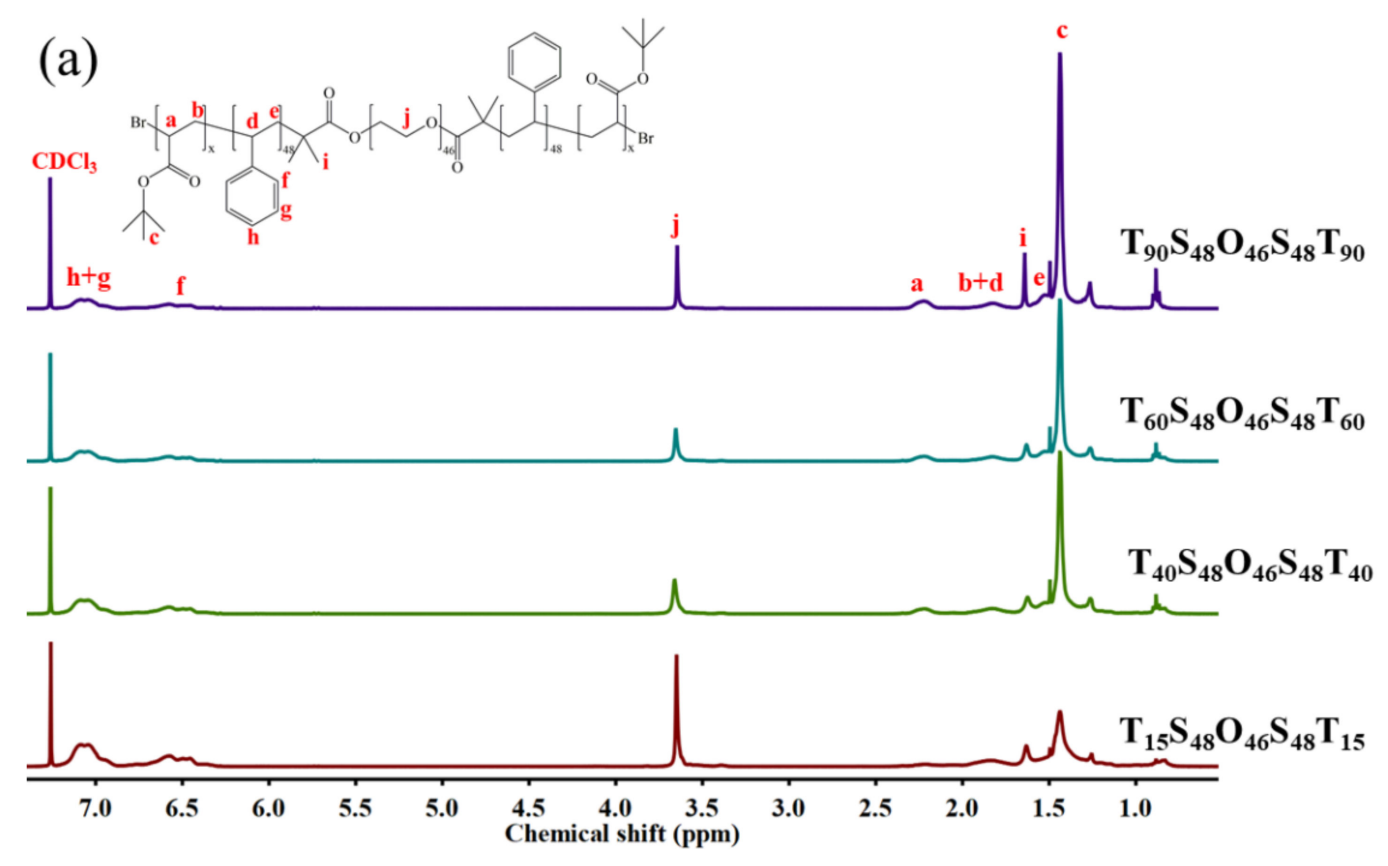

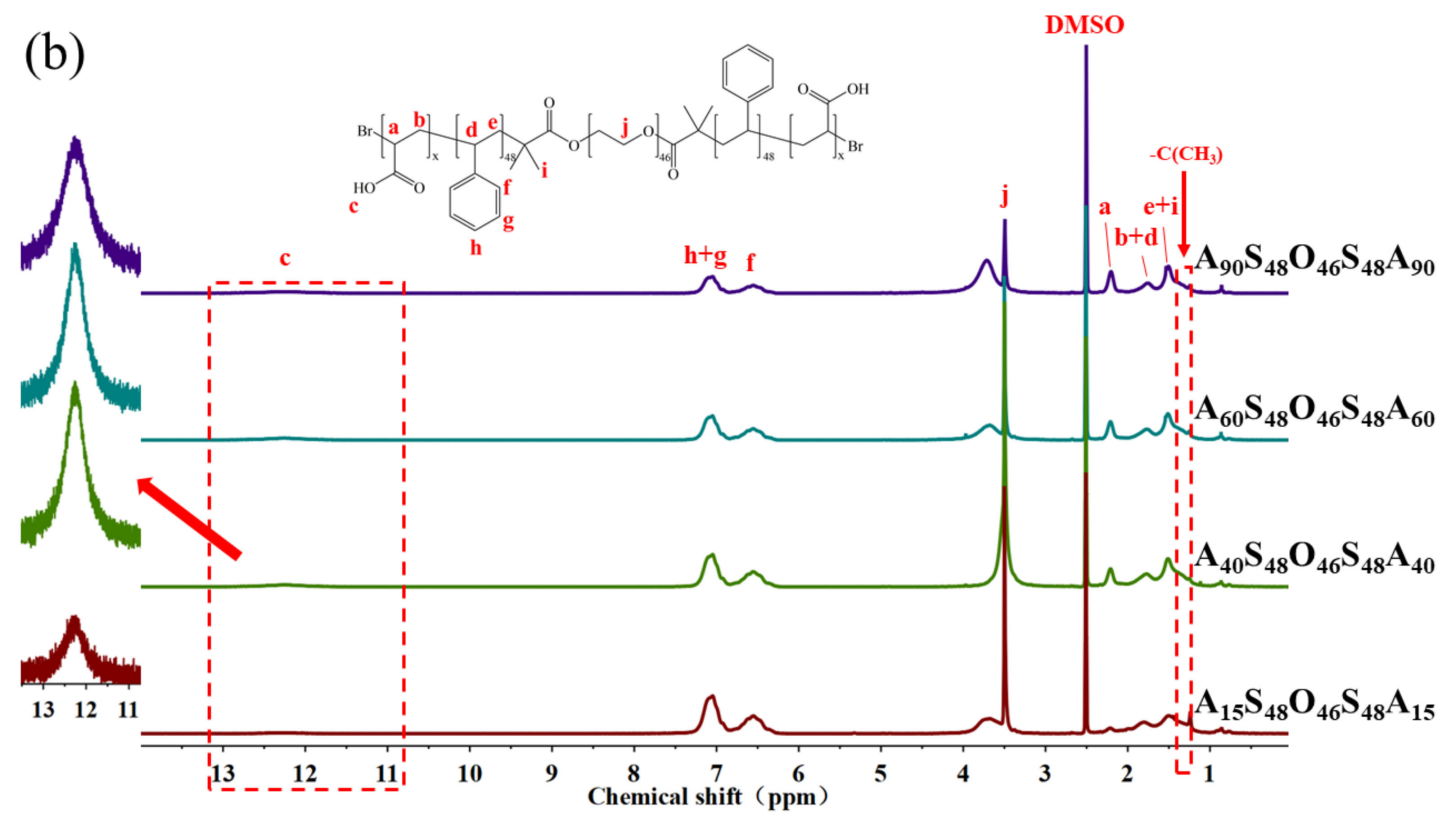
^1^H NMR spectra of (**a**) T*_x_*S_48_O_46_S_48_T*_x_* (*x* = 15, 40, 60, 90) in CDCl_3_ and (**b**) A*_x_*S_48_O_46_S_48_A*_x_* (*x* = 15, 40, 60, 90) in DMSO-*d_6_*.

**Figure 2:**
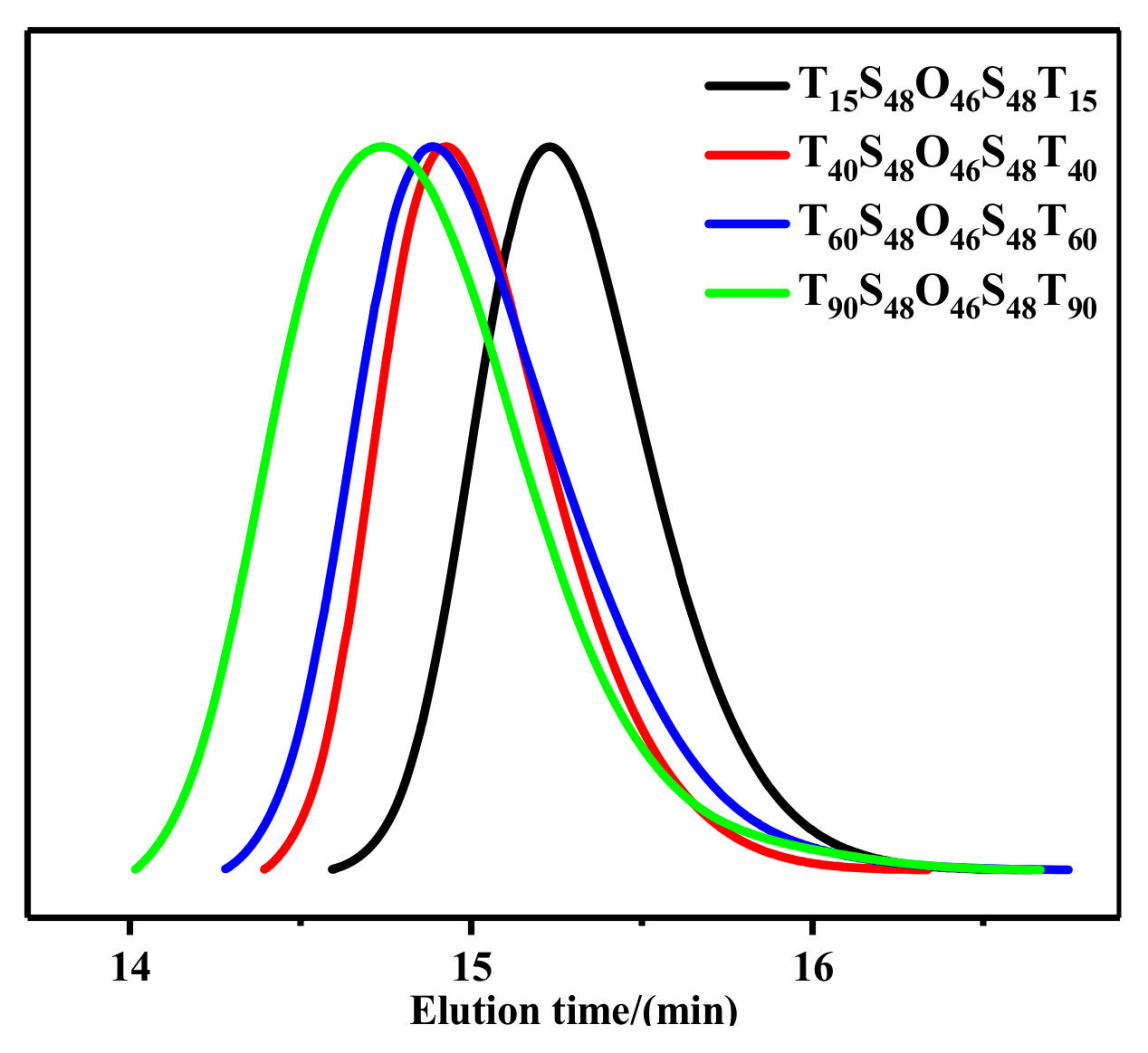
GPC traces of T*_x_*S_48_O_46_S_48_T*_x_* (*x* = 15, 40, 60, 90) pentablock terpolymers.

A*_x_*S_48_O_46_S_48_A*_x_* pentablock terpolymers with different lengths *x* of PAA blocks were synthesized via a two-step ATRP process with Br-PEO_46_-Br as the macroinitiator, St as the first monomer, and *t*BA as the second monomer, followed with the hydrolysis of P*t*BA blocks, as shown in Scheme 1. Four A*_x_*S_48_O_46_S_48_A*_x_* pentablock terpolymers with *x* = 15, 40, 60, and 90 were obtained in the present work. Figure 1 shows the ^1^H NMR spectra of A*_x_*S_48_O_46_S_48_A*_x_* and corresponding parent T*_x_*S_48_O_46_S_48_T*_x_* pentablock terpolymers. The characteristic peak of –C(CH_3_)_3_ with chemical shift of ca. 1.35 ppm, which can be clearly observed for T*_x_*S_48_O_46_S_48_T*_x_* (Figure 1a), completely disappeared for A*_x_*S_48_O_46_S_48_A*_x_* (Figure 1b), indicating that the P*t*BA blocks were completely hydrolyzed and turned into PAA blocks. The degrees of polymerization of S (48) and T (*x*) blocks were determined from the ^1^H NMR spectra using the known value of the O block (46). Figure 2 shows the GPC traces of four T*_x_*S_48_O_46_S_48_T*_x_* pentablock polymers, which exhibited narrow dispersities (*M_w_/M_n_s* < 1.20). Note that it was difficult to determine the *M*_w_/*M*_n_s of A*_x_*S_48_O_46_S_48_A*_x_* using GPC because of the strong polarity of the PAA block, which would be strongly adsorbed onto the GPC column during the GPC measurement, leading to the wrong results of molecular weight and dispersity. The *M*_w_/*M*_n_s of T*_x_*S_48_O_46_S_48_T*_x_* were used to present the *M*_w_/*M*_n_s of A*_x_*S_48_O_46_S_48_A*_x_*, which were expected to be in the same range as that of the corresponding T*_x_*S_48_O_46_S_48_T*_x_*, as discussed in our previous work [36]. Table 1 summarizes the number-average molecular weight (*M*_n_) and dispersity (*M_w_/M_n_*) of A*_x_*S_48_O_46_S_48_A*_x_* pentablock terpolymers studied in the present work.

### 3.2. Morphologies and Structures of A_x_S_48_O_46_S_48_A_x_ Micelles in Dilute Aqueous Solutions

**Figure 3:**
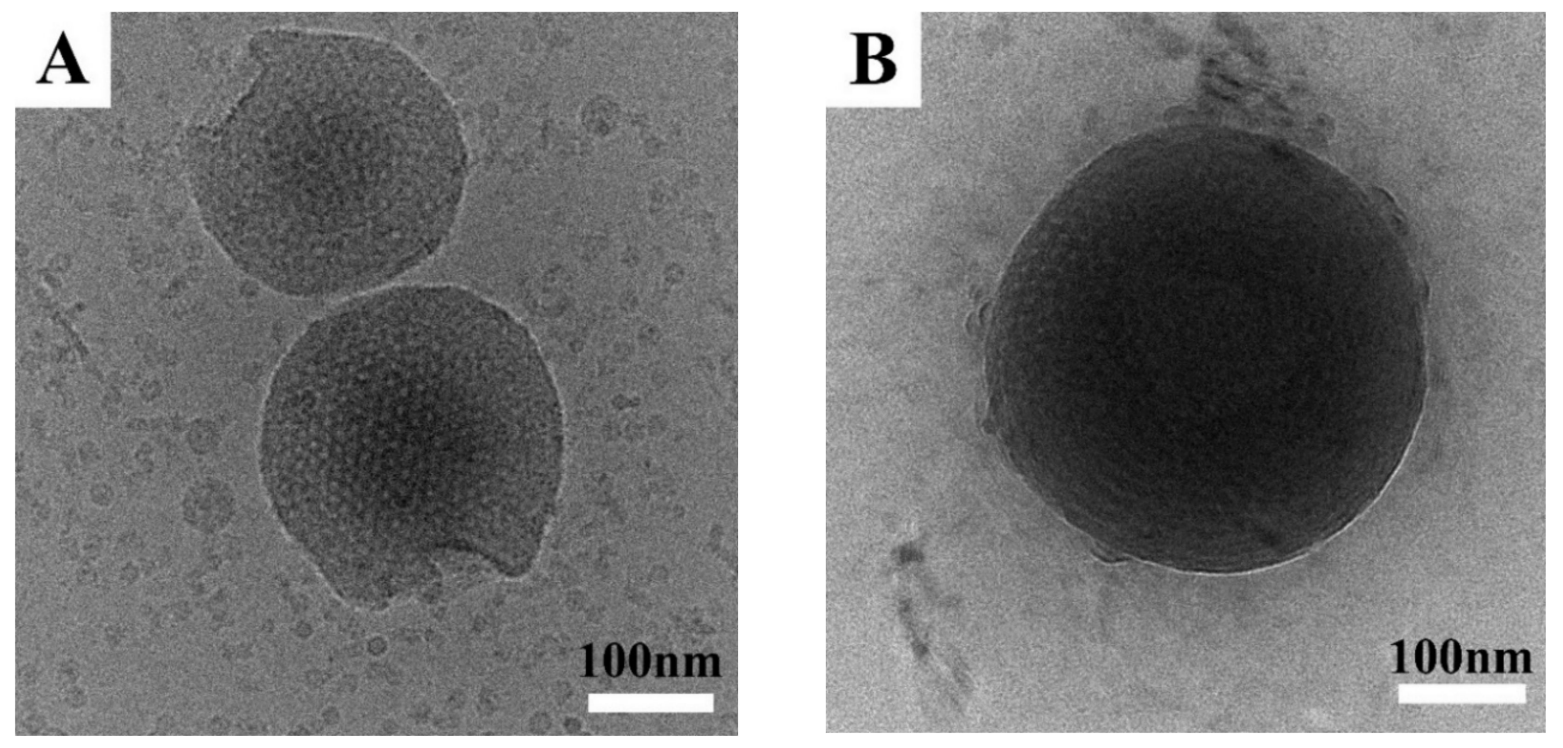

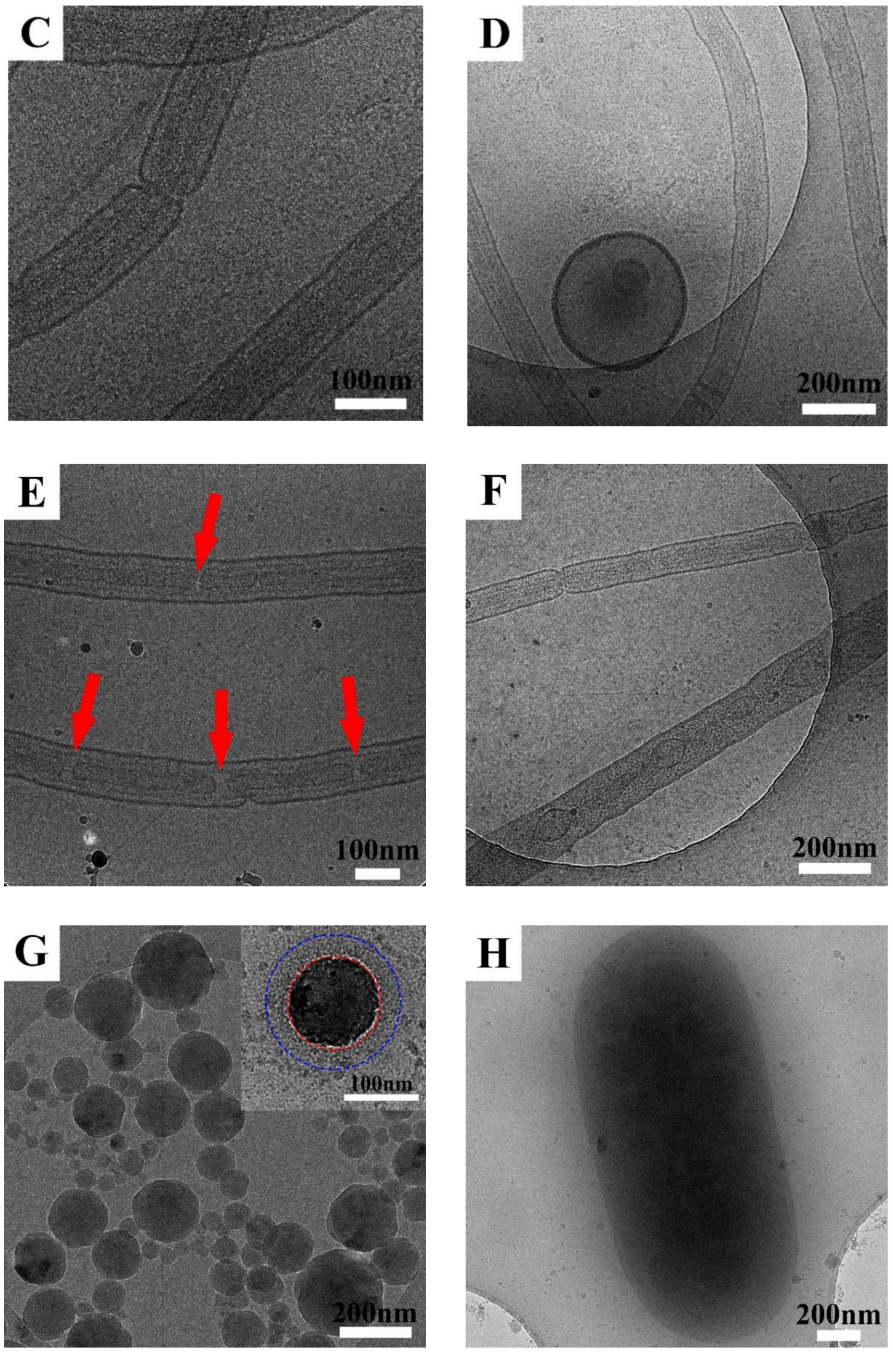
Cryo-TEM images of (**A**) and (**B**) A_15_S_48_O_46_S_48_A_15_, (**C**) and (**D**) A_40_S_48_O_46_S_48_A_40_, (**E**) and (**F**) A_60_S_48_O_46_S_48_A_60_, (**G**) and (**H**) A_90_S_48_O_46_S_48_A_90_ micelles in dilute aqueous solutions. The red arrows in Figure 3E point out the fractures of inner nanotubes.

**Scheme 2:**
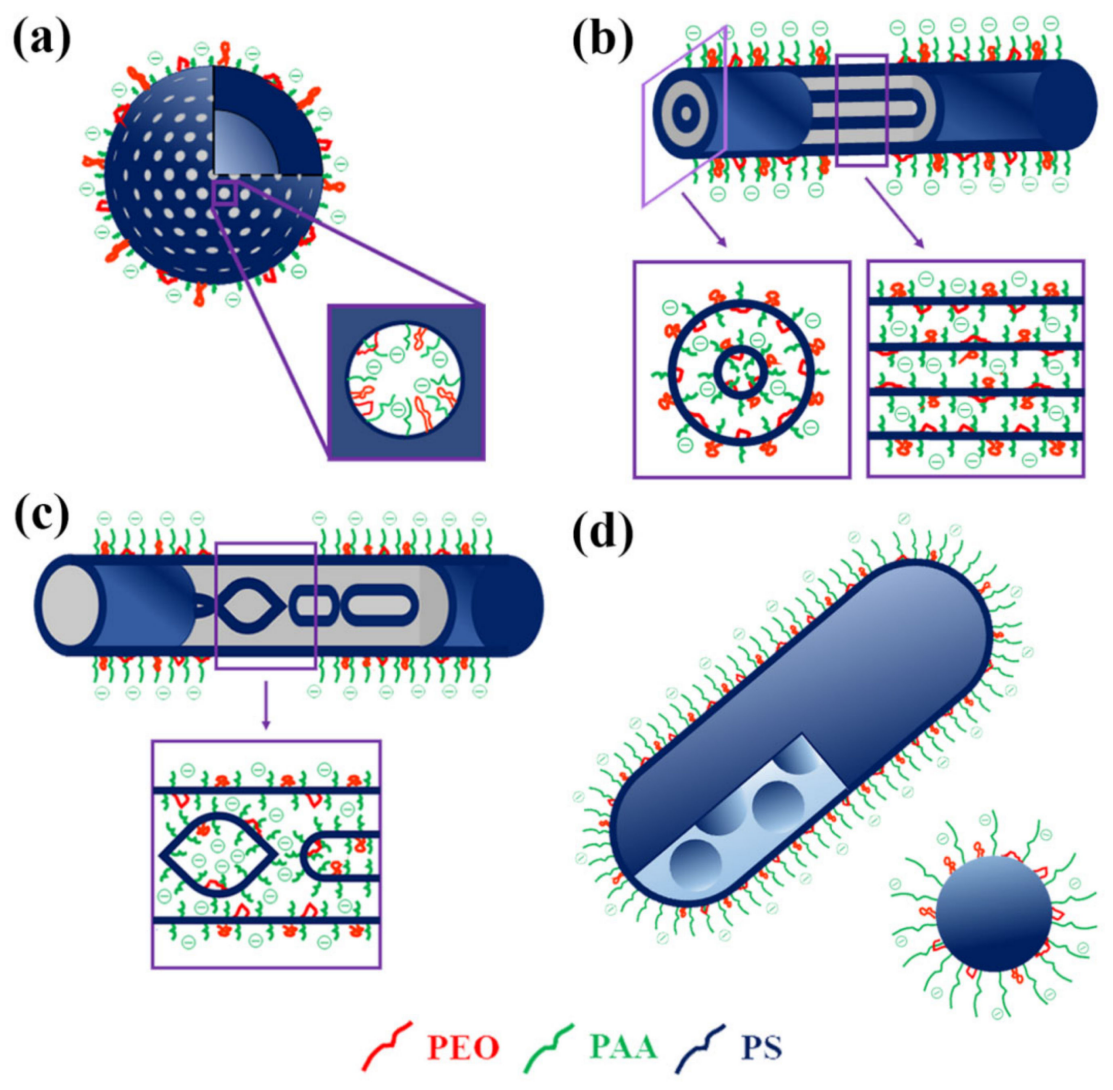
The schematic morphologies and possible chain structures of the observed A*_x_*S_48_O_46_S_48_A*_x_* micelles in dilute aqueous solutions. (**a**) *x* = 15, (**b**) *x* = 40, (**c**) *x* = 60, (**d**) *x* = 90.

Because of the amphiphilic nature of A*_x_*S_48_O_46_S_48_A*_x_* pentablock terpolymers, they formed micelles in dilute aqueous solutions. Note that the PS block is hydrophobic and the PEO and PAA blocks are hydrophilic. Figure 3 shows the representative cryo-TEM images of A_15_S_48_O_46_S_48_A_15_, A_40_S_48_O_46_S_48_A_40_, A_60_S_48_O_46_S_48_A_60_, and A_90_S_48_O_46_S_48_A_90_ micelles in dilute aqueous solutions. Additional cryo-TEM images were also given in Figure S2. The observed morphologies were well reproducible. It can be seen that A_15_S_48_O_46_S_48_A_15_ pentablock terpolymers formed spherical vesicles with ordered porous membranes in aqueous solutions, which were rarely reported for block copolymer micelles (Figure 3A,B and Figure S2A). The preparation of porous vesicles usually required sophisticated design of polymer chains or a complicated preparation process. Kim et al. [37] reported the fabrication of porous supramolecular microcapsules by using specially designed dumbbell-shaped rod amphiphiles, which consisted of a phenylacetylene segment in the middle with hydrophilic polyether dendrons and hydrophobic alkane branches on each side. Xu et al. [38] reported the fabrication of polystyrene-*b*-poly(4-vinylpyridine) (PS-*b*-P4VP) block copolymer vesicles with porous membranes by using an emulsion solvent evaporation method with a high-boiling non-solvent droplet as the liquid core template. By varying the block length and ratio of PS and P4VP blocks as well as the additives, the spherical, toroid, and onion mesopores in the membranes were obtained for the PS-*b*-P4VP vesicles. Wong et al. [39] prepared the vesicles with inverse hexagonal pores in the membranes with poly(glycosyloxyethyl methacrylate)-*b*-poly(benzyl acrylate)-*b*-poly(4-vinylpyridine) triblock terpolymer (PGlcEMA-*b*-PBzA-*b*-P4VP) by liquid–liquid phase separation and polymer microphase separation processes. First, PGlcEMA-*b*-PBzA-*b*-P4VP was dissolved in DMF/MeOH mixed solutions and formed polymer/DMF-rich droplets dispersed in MeOH mixture, where DMF was the good solvent and MeOH was the poor solvent for all three blocks. During the dialysis against MeOH, the PGlcEMA-*b*-PBzA-*b*-P4VP chains migrated to the interface and formed the membranes to stabilize the droplets, and ultimately, underwent phase separation into a three-layer inverse hexagonal phase when DMF content continuously decreased. Herein, vesicles with ordered porous vesicles were obtained by directly dialyzing A_15_S_48_O_46_S_48_A_15_ THF solution against deionized water. From Figure 3A, partial collapse of membranes was clearly observed, which was a typical phenomenon for vesicles and resulted from the pressure difference between the interior and exterior of the vesicles during sample preparation for TEM observation [1]. The diameters of A_15_S_48_O_46_S_48_A_15_ vesicles were about 200–300 nm and the diameters of pores in the vesicle membranes were about 7 nm. The hydrophobic PS blocks formed the walls of vesicles and the hydrophilic PEO and PAA blocks on the membrane surfaces stabilized the vesicles, as illustrated in Scheme 2. Increasing the length *x* of PAA blocks from 15 to 40, the long double nanotubes were mostly observed for A_40_S_48_O_46_S_48_A_40_ aqueous solutions (Figure 3C,D and Figure S2B). Interestingly, the inner nanotubes were almost exactly located in the middle of outer nanotubes. The diameter of the outer nanotube was about 95 nm and the diameter of the inner nanotube was about 34 nm. The wall thicknesses of the outer and inner nanotubes were almost the same, i.e., about 7 ± 0.8 nm. The gap between the outer nanotubes and inner nanotubes was about 24 nm. The walls of the nanotubes were composed of PS blocks, which appeared dark in cryo-TEM images. Similarly, the hydrophilic PEO and PAA blocks on the surface of nanotubes stabilized the long double nanotubes in aqueous solutions. Because of the low molecular weight (5000 g/mol), the PEO block cannot be observed in cryo-TEM images [40]. The PAA chains were also invisible as they were highly swollen in aqueous solution [41] and also, too short to be identified. Such double nanotubes were, to our best knowledge, the first reported for block copolymer micelles in aqueous solutions. Besides the long double nanotubes, a few spherical vesicles were also observed for A_40_S_48_O_46_S_48_A_40_ (Figure 3D). When the length *x* of the PAA block increased to 60, the long nanotubes were observed for A_60_S_48_O_46_S_48_A_60_ aqueous solutions (Figure 3E,F and Figure S2C,D). However, these long nanotubes were different with those observed for A_40_S_48_O_46_S_48_A_40_. The diameter of the outer nanotube increased to 110~130 nm and the inner nanotubes ruptured into many short nanotubes. Modulation of inner short nanotubes was also observed (Figure 3F). The wall thicknesses of outer long nanotubes and inner short nanotubes or modulated nanotubes were the same as those of A_40_S_48_O_46_S_48_A_40_ nanotubes, i.e., about 7 nm. Expansion and distortion of the nanotubes were also observed, as shown in Figure S2C,D. Possibly, the increased length of the PAA block resulted in stronger repulsion interactions between the outer and inner nanotubes, causing the rupture of the inner nanotubes. The extended chain lengths (*R*_E_) of PEO and PAA blocks can be estimated by using Equation (1), given as [35]:

(1) $$R_{E}=nlsin\left(\frac{\theta }{2}\right)$$

where *n* is the degree of polymerization of block chain, *l* is the length of covalent bond, and *θ* is the angle of bonds. The lengths *l* of C–C and C–O bonds are 0.154 and 0.143 nm, respectively. The angle *θ* of C–C–C and C–O–C are 109.5° and 108°, respectively. *R*_E_ of the PEO block was then calculated to be about 16 nm, which was the same for A_40_S_48_O_46_S_48_A_40_ and A_60_S_48_O_46_S_48_A_60_. As a result, the longest length of the hydrophilic PEO block on the surface of nanotubes was about 8 nm because the PEO block was located in the middle of A_40_S_48_O_46_S_48_A_40_ and A_60_S_48_O_46_S_48_A_60_ pentablock terpolymers and had to fold during the formation of nanotube micelles. For A_40_S_48_O_46_S_48_A_40_, *R*_E_ of each PAA block was about 10 nm. From Figure 3C,D, the gap between the outer nanotubes and inner nanotubes was about 24 nm, which was larger than twice of *R*_E_ of the PAA or PEO block. In other words, the hydrophilic PEO and PAA block chains on the surfaces of outer and inner nanotubes did not touch each other for the long double nanotubes of A_40_S_48_O_46_S_48_A_40_. However, *R*_E_ of the PAA block increased to about 16 nm for A_60_S_48_O_46_S_48_A_60_. The gap of 24 nm between the outer and inner nanotubes was less than twice of *R*_E_ of the PAA block, i.e., about 32 nm, which meant that the hydrophilic PAA block chains on the surface of outer and inner nanotubes would touch each other. As a result, the size of outer nanotubes increased, and the inner nanotubes ruptured to form short tubes in order to avoid such touch and release the energy penalty for A_60_S_48_O_46_S_48_A_60_. Further increasing the length of the PAA block to *x* = 90, spherical micelles and some large compound vesicles with spherical micelles inside were observed for A_90_S_48_O_46_S_48_A_90_ aqueous solutions, as shown in Figure 3G,H. The diameters of the spherical micelles were in the range of 100–200 nm. The corona chains of spherical micelles can be clearly observed from the magnified cryo-TEM image shown in Figure S2E and the inset of Figure 3G. The length of corona chain was measured to be about 20 nm, which was slightly smaller than the extended chain length *R*_E_ of the PAA block, i.e., about 23 nm. The sizes of large compound vesicles were about 700–800 nm and the sizes of spherical micelles inside the vesicles were about 100–200 nm. More large compound vesicles were shown in Figure S2F. Probably, these large compound vesicles were the intermediate between the long nanotubes observed for A_60_S_48_O_46_S_48_A_60_ and the spherical micelles shown in Figure 3G. The rupture of the large compound vesicles would release the inside spherical micelles.

### 3.3. Dynamic Light Scattering Measurements of A_x_S_48_O_46_S_48_A_x_ Micelles in Dilute Aqueous Solutions

**Figure 4:**
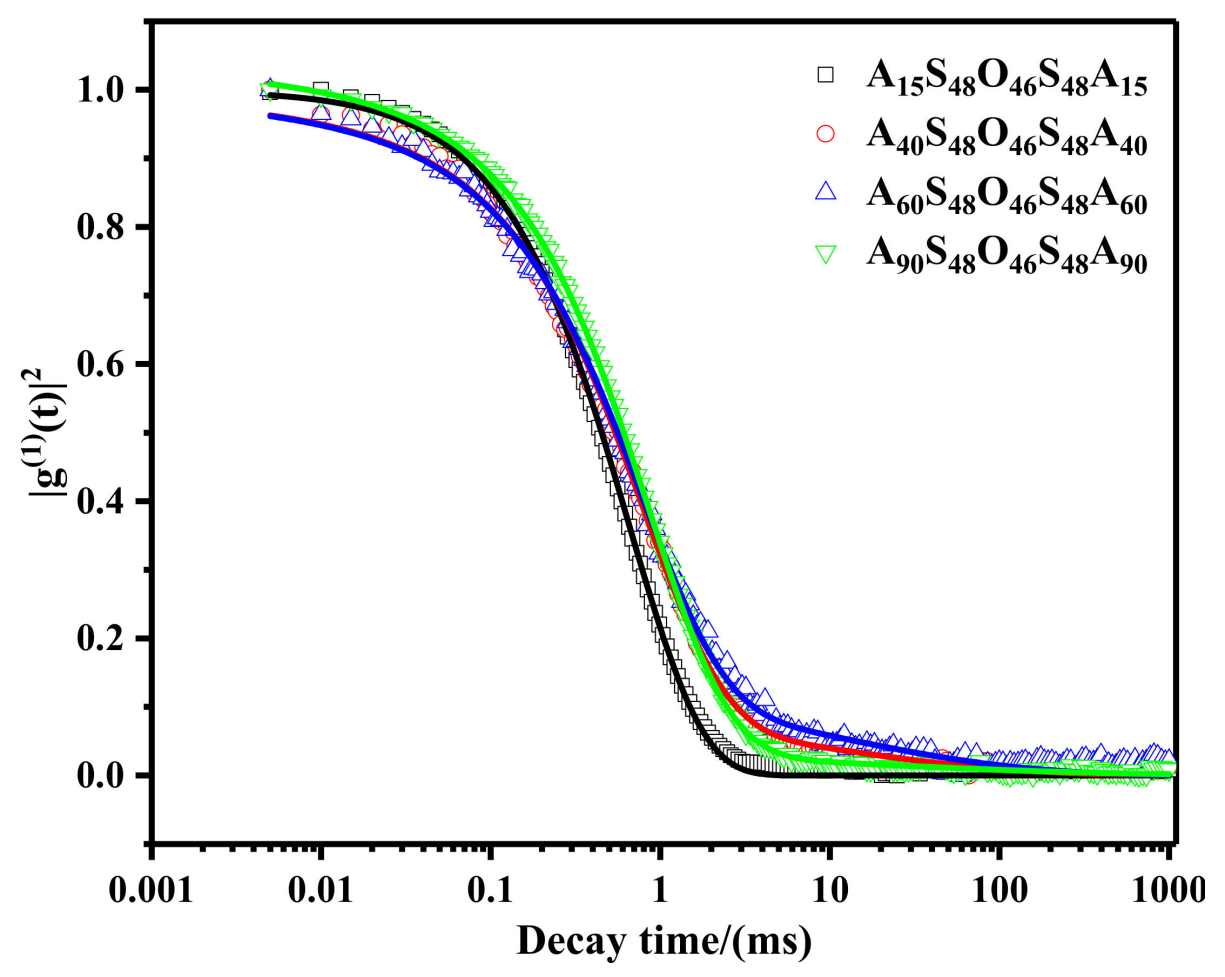
Normalized electric field autocorrelation function g^(1)^(*t*) of A*_x_*S_48_O_46_S_48_A*_x_* micelles (*x* = 15, 40, 60, 90) in dilute aqueous solutions measured by DLS at 25 °C and scattering angle *θ* of 90°. The solid lines well fit with Equation (2) with *R*^2^ ≥ 0.999.

The self-assembly behavior of A*_x_*S_48_O_46_S_48_A*_x_* micelles in dilute aqueous solutions was investigated by dynamic light scattering (DLS). Figure 4 shows the normalized electric field autocorrelation functions *g*^(1)^(*t*) of A*_x_*S_48_O_46_S_48_A*_x_* micelles in dilute aqueous solution at 25 °C and scattering angle *θ* of 90°. These decay curves of *g*^(1)^(*t*) can be described by using a single exponential decay function plus a stretched exponential function given as [42,43]:

(2) $$g^{\left(1\right)}(t)\;=\;A_{\mathrm{fast}}\exp\left(-\frac{t}{\tau_{\mathrm{fast}}}\right)+A_{\mathrm{slow}}\exp\left[-{\left(\frac{t}{\tau_{\mathrm{slow}}}\right)}^{\gamma }\right]$$

where the pre-factors *A*_fast_ and *A*_slow_ are the amplitudes of the fast and slow relaxation modes, respectively, and the sum of *A*_fast_ and *A*_slow_ is close to 1. τ_fast_ and τ_slow_ represent the relaxation times of the fast and slow modes, respectively. The reciprocal of *τ* is the relaxation rate *Γ* (*Γ* = 1/*τ*). The stretched exponent, γ, (0 < γ ≤ 1) is inversely proportional to the width of the distribution of the relaxation time. A larger value of γ implies a narrower distribution of the relaxation time. Equation (2) has been widely used to describe micelles with anisotropic shapes in solutions. Fast and slow relaxation modes have been applied to distinguish the difference of motion modes for the solutions containing objects with anisotropic shapes or with diverse sizes and morphologies [42,44,45]. The fitting results were summarized in Table 2.

It can be seen that g^(1)^(*t*) of A_15_S_48_O_46_S_48_A_15_ micelles in dilute aqueous solution can be well described by a single exponential decay function, which indicated that the A_15_S_48_O_46_S_48_A_15_ micelles were spherical in shape with relatively narrow size distribution. This result was consistent with the observation of cryo-TEM for A_15_S_48_O_46_S_48_A_15_ micelles, as shown in Figure 3A. The hydrodynamic radius (*R*_h_) of A_15_S_48_O_46_S_48_A_15_ micelles can be estimated from τ_fast_ value by using the Stokes–Einstein equation:

(3) $$R_{h}=\frac{k_{B}T}{6\pi \eta D}$$

where *k*_B_ is the Boltzmann constant (1.38 × 10^−23^ J/K), *T* is the Kelvin temperature, *η* is the viscosity of water at 25 °C, i.e., 0.89 × 10^−3^ N·s·m^−2^, and *D* = 1/(*q*^2^ × τ_fast_) is the mutual diffusion coefficient of the micelles in solution with scattering vector *q*. The scattering vector *q* can be calculated by $q=\frac{4\pi n}{\lambda }sin\frac{\theta }{2}$, where *n* is the refractive index of the solution, λ is the wavelength of laser light in vacuum, and *θ* is the scattering angle. The value of *R*_h_ was calculated to be about 110 nm, which was in good agreement with those obtained from cryo-TEM images, i.e., 100–150 nm. For A_40_S_48_O_46_S_48_A_40_ and A_60_S_48_O_46_S_48_A_60_ micelles in dilute aqueous solutions, g^(1)^(*t*)s were well fitted by Equation (2) with stretched exponential function, indicating the existence of anisotropic micelles in solutions. The fitting values of *A*_fast_, *A*_slow_, and γ were similar for A_40_S_48_O_46_S_48_A_40_ and A_60_S_48_O_46_S_48_A_60_ micelles, suggesting that the micelle morphologies and size distributions of the two samples were similar. The slight increase in *A*_fast_ and τ_fast_ of A_40_S_48_O_46_S_48_A_40_ micelles might be attributed to the existence of small spherical vesicles in the solutions as showed in Figure 3D, which were not observed for A_60_S_48_O_46_S_48_A_60_ micelle solutions. Furthermore, τ_slow_ of A_60_S_48_O_46_S_48_A_60_ micelles was slightly larger than that of A_40_S_48_O_46_S_48_A_40_ micelles, which might be attributed to the larger diameter of the long outer nanotubes and the modulation of nanotubes, as shown in Figure 3E,F and Figure S2C,D. For A_90_S_48_O_46_S_48_A_90_ micelle solutions, the hydrodynamic radius estimated from τ_fast_ was about 125 nm, which was slightly larger than the size of spherical micelles but much smaller than the size of large compound vesicles obtained by cryo-TEM. The slow mode presented by τ_slow_ could be attributed to the large compound vesicles with spherical micelles inside. *A*_fast_ was much larger than *A*_slow_, suggesting that the spherical micelles were the majority in A_90_S_48_O_46_S_48_A_90_ micelle solutions. Furthermore, the small value of γ suggested a board size distribution of micelles, which was consistent with the cryo-TEM observation shown in Figure 3G,H.

### 3.4. Possible Chain Structure of A_x_S_48_O_46_S_48_A_x_ Micelles in Dilute Aqueous Solutions

**Figure 5:**
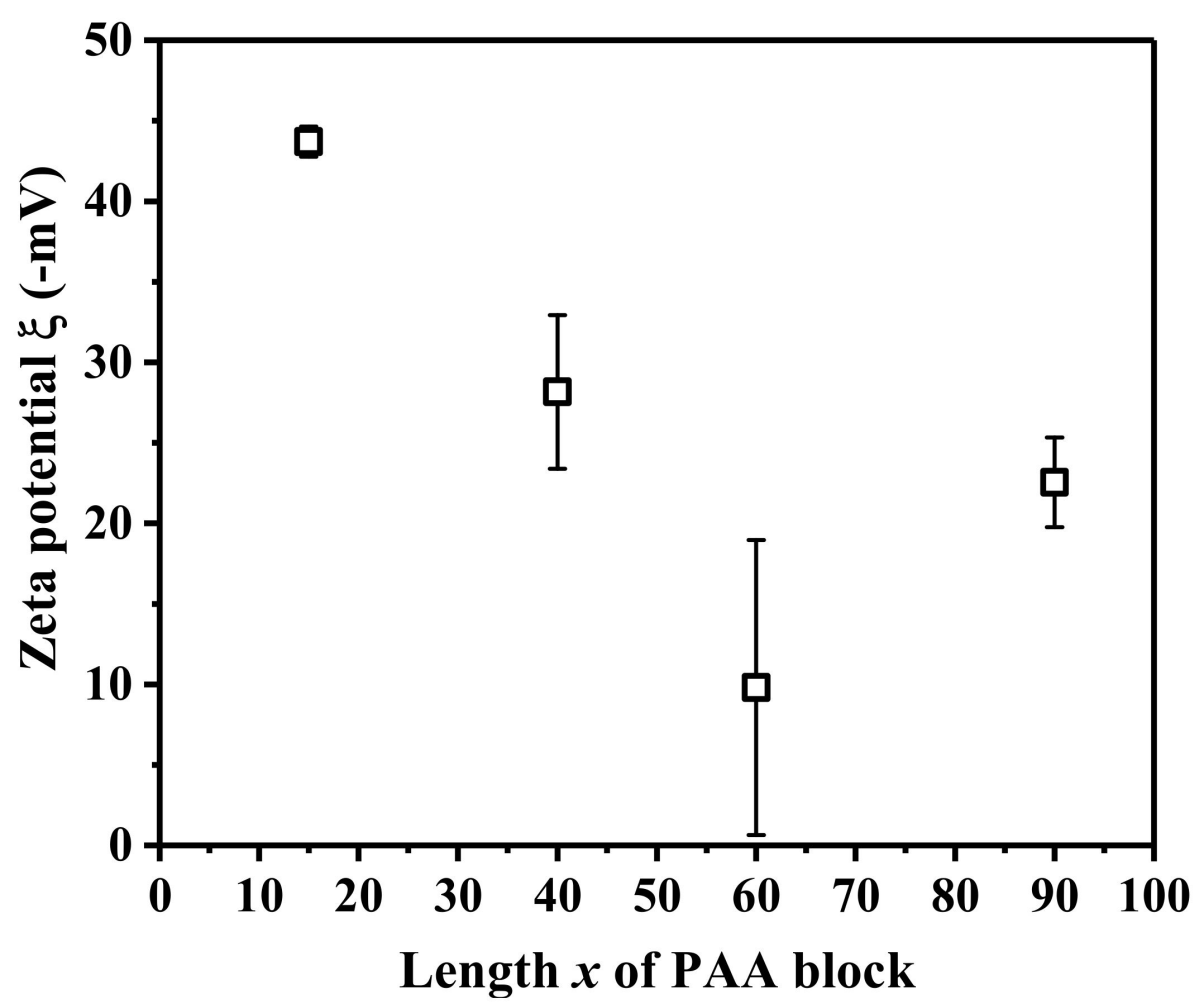
Zeta potential ξ of A*_x_*S_48_O_46_S_48_A*_x_* with various length *x* of PAA block (*x* = 15, 40, 60, 90) micelles in aqueous solutions at 25 °C.

For A*_x_*S_48_O_46_S_48_A*_x_* micelles in dilute aqueous solutions, spherical vesicles with ordered porous membranes, a long double nanotube, a long nanotube with an inner modulated nanotube or short nanotube, and spherical micelles or large compound vesicles with spherical micelles inside were observed for A_15_S_48_O_46_S_48_A_15_, A_40_S_48_O_46_S_48_A_40_, A_60_S_48_O_46_S_48_A_60_, and A_90_S_48_O_46_S_48_A_90_ in dilute aqueous solutions, respectively, with increasing the length *x* of the PAA block. Scheme 2 shows the schematic morphologies and possible chain structures of the observed A*_x_*S_48_O_46_S_48_A*_x_* micelles. The hydrophobic PS blocks formed the walls of vesicles and nanotubes as well as the core of spherical micelles. The hydrophilic PEO and PAA block chains were on the surfaces of vesicle membranes, nanotubes, and spherical micelles, stabilizing the micelles in aqueous solutions. The middle PEO block chain also folded because of the chain connection restriction. PAA is a weak polyanion with pKa of ~5 and the ionization degree of the PAA chain is strongly dependent on the pH value of solutions [46,47]. In deionized water, the PAA chains are partly ionized. Figure 5 shows the zeta potential ξ of A*_x_*S_48_O_46_S_48_A*_x_* micelles in dilute aqueous solutions. The negative value of ξ indicated that the PAA block chains were on the surfaces of the micelles and ionized in aqueous solutions. The value of ξ for block polymer micelles in dilute aqueous solutions was affected by the pH of solutions [48], the nanostructure of micelles, and the density of charged block chains on the outer surfaces of micelles [49]. Therefore, the values of ξ of A*_x_*S_48_O_46_S_48_A*_x_* micelles in dilute aqueous solutions were related with the morphologies and chain structures of the micelles. It can be seen from Figure 5 that the absolute value of ξ first decreased from about 43.7 to 9.8 mV when increasing PAA block length *x* from 15 to 60 and then, increased again to 22.5 mV for *x* of 90. ξ of −43.7 mV revealed large amounts of ionized PAA chains located on the outer surfaces of A_15_S_48_O_46_S_48_A_15_ vesicles. With increasing *x* to 40 and 60, the long double nanotubes were formed for A_40_S_48_O_46_S_48_A_40_ and A_60_S_48_O_46_S_48_A_60_, causing the reduction in specific surface area and the decrease in ionized PAA chains on the outer surfaces of the outer nanotubes. Furthermore, the ionized PAA chains on the surface of inner nanotubes did not contribute to the value of ξ. For A_40_S_48_O_46_S_48_A_40_ micelles, the remaining vesicles besides nanotubes result in a larger variation of the Zeta potential. On the other hand, for A_60_S_48_O_46_S_48_A_60_ micelles, the deformations, fractures, and knots of nanotubes make the error bar of ξ even broader. For A_90_S_48_O_46_S_48_A_90_ micelles with *x* of 90, ξ increased again to about 22.5 mV. The formation of spherical micelles increased the corona surface area where the ionized PAA chains were located. However, ξ of A_90_S_48_O_46_S_48_A_90_ micelles was still smaller than that of A_15_S_48_O_46_S_48_A_15_ vesicles, which might be attributed to the formation of large compound vesicles with spherical micelles inside. Similarly, the spherical micelles inside the vesicles did not contribute to the value of ξ. Furthermore, the coexistence of different micelle structures and containing micelles in micelles might result in a large error bar when determining ξ for A_40_S_48_O_46_S_48_A_40_, A_60_S_48_O_46_S_48_A_60_, and A_90_S_48_O_46_S_48_A_90_ micelles in aqueous solutions.

## 4. Conclusions

In summary, four A*_x_*S_48_O_46_S_48_A*_x_* pentablock terpolymers with different lengths *x* of PAA block (*x* = 15, 40, 60, and 90) were synthesized via a two-step ATRP process with Br-PEO_46_-Br as the macroinitiator, St as the first monomer, and *t*BA as the second monomer, followed with the hydrolysis of P*t*BA blocks. The A*_x_*S_48_O_46_S_48_A*_x_* pentablock terpolymers formed micelles with various morphologies in dilute aqueous solutions depending on the length *x* of the PAA block. The morphology of A*_x_*S_48_O_46_S_48_A*_x_* micelles changes from spherical vesicles with ordered porous membranes to long double nanotubes, then to long nanotubes with inner modulated nanotubes or short nanotubes, and finally, to spherical micelles or large compound vesicles with spherical micelles inside with increasing the length *x* of the PAA block from 15 to 90. The hydrophobic PS blocks formed the walls of vesicles and nanotubes as well as the core of spherical micelles. The hydrophilic PEO and partially ionized the PAA block chains located on the surfaces of vesicle membranes, nanotubes, and spherical micelles, leading to the negative zeta potential of the A*_x_*S_48_O_46_S_48_A*_x_* micelles and stabilizing the micelles in dilute aqueous solutions. The porous vesicles and double layer nanotubes might have potential applications as drug delivery and material transporting media.

## Figures and Tables

**Table 1:** Number-average molecular weight, dispersity, volume fraction, and micelle morphology of A*_x_*S_48_O_46_S_48_A*_x_* pentablock terpolymers.

Sample	*M_n_^a^* (kg/mol)	*M*_w_ */M*_n_ *^b^*	Morphology *^c^*
A_15_S_48_O_46_S_48_A_15_	14.2	1.10	vesicles with ordered porous membrane
A_40_S_48_O_46_S_48_A_40_	17.8	1.11	long double nanotubes
A_60_S_48_O_46_S_48_A_60_	20.7	1.15	long double nanotubes with modulated inner nanotube
A_90_S_48_O_46_S_48_A_90_	25.0	1.19	spherical micelles and large compound vesicles

*^a^ M*_n_ is the number-average molecular weight determined by ^1^H NMR.*^b^ M*_w_/*M*_n_ is the dispersity of corresponding T*_x_*S_48_O_46_S_48_T*_x_* pentablock terpolymers determined by GPC. ^c^ Determined by cryo-TEM.

**Table 2:** The fitting parameters of Equation (2) for g^(1)^(*t*) of A*_x_*S_48_O_46_S_48_A*_x_* micelles in dilute aqueous solutions at 25 °C and scattering angle of 90°.

Sample	A_15_S_48_O_46_S_48_A_15_	A_40_S_48_O_46_S_48_A_40_	A_60_S_48_O_46_S_48_A_60_	A_90_S_48_O_46_S_48_A_90_
*A*_fast_	1	0.637	0.577	0.782
*A*_slow_	0	0.363	0.423	0.246
τ_fast_ (ms)	1.3	1.283	1.211	1.511
τ_slow_ (ms)	—	44.845	52.711	99.265
γ	—	0.338	0.345	0.246

## Supplementary Materials

The following are available online at https://www.mdpi.com/2073-4360/12/10/2183/s1. Figure S1. ^1^H NMR spectrum of Br-PEO_46_-Br macroinitiator. Figure S2. Additional cryo-TEM images of (A) A_15_S_48_O_46_S_48_A_15_, (B) A_40_S_48_O_46_S_48_A_40_, (C and D) A_60_S_48_O_46_S_48_A_60_, (E and F) A_90_S_48_O_46_S_48_A_90_ micelles in dilute aqueous solutions.
