# Supplementary material for: Self-Assembly of Linear Amphiphilic Pentablock Terpolymer PAAx-PS48-PEO46-PS48-PAAxin Dilute Aqueous Solution
Source: Polymers (Basel). 2020 Sep 24;12(10):2183. doi: 10.3390/polym12102183 (PMC7598608; doi:10.3390/polym12102183)
Supplement: Supplementary file 1 [file polymers-12-02183-s001.pdf]

Self-Assembly of Linear Amphiphilic Pentablock Terpolymer PAA$_x$-PS$_{48}$-PEO$_{46}$-PS$_{48}$-PAA$_x$ in Dilute Aqueous Solution

Jia Gao [1], Kun An [1], Chao Lv [1], Jingjing Nie [2], Junting Xu [1], and Binyang Du [1,*]

[1] MOE Key Laboratory of Macromolecular Synthesis and Functionalization, Department of Polymer Science & Engineering, Zhejiang University, Hangzhou 310027, China; 11629010@zju.edu.cn (J.G.); 21929006@zju.edu.cn (K.A.); 21629025@zju.edu.cn (C.L.); xujt@zju.edu.cn (J.X.)

[2] Department of Chemistry, Zhejiang University, Hangzhou 310027, China; niejj@zju.edu.cn (J.N.)

* Correspondence: duby@zju.edu.cn (B.D.)

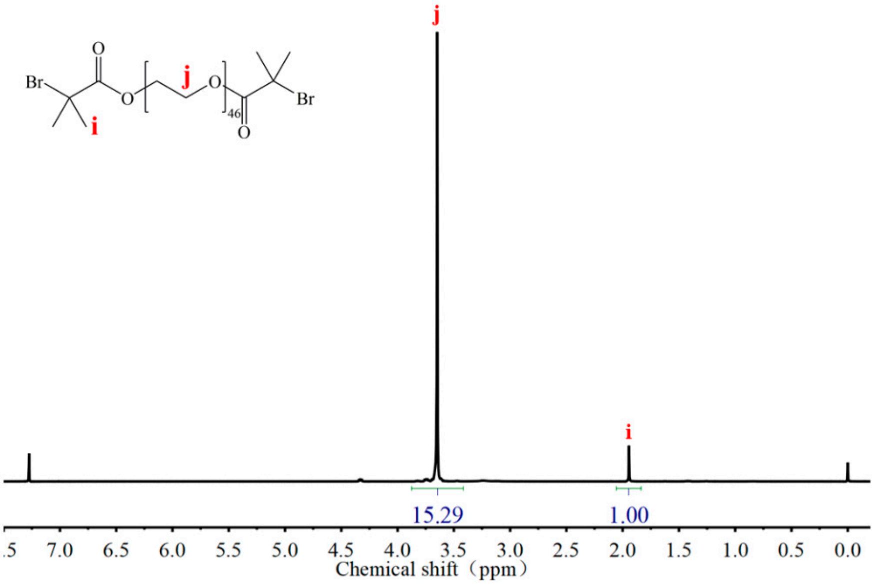

Figure S1. ^{1}H NMR spectrum of Br-PEO$_{46}$-Br macroinitiator.

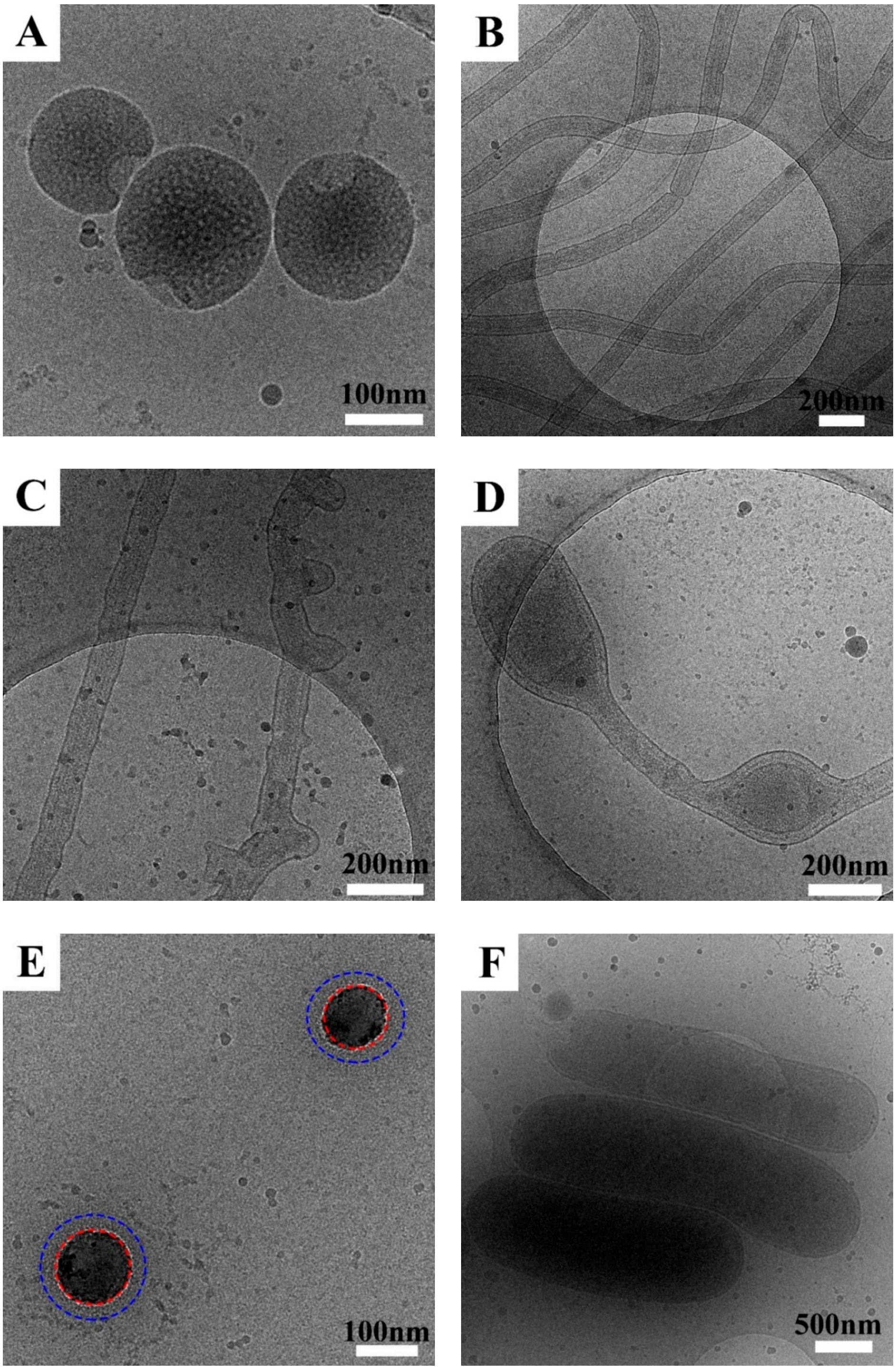

Figure S2. Additional cryo-TEM images of (A) $A_{15}S_{48}O_{46}S_{48}A_{15}$, (B) $A_{40}S_{48}O_{46}S_{48}A_{40}$, (C and D) $A_{60}S_{48}O_{46}S_{48}A_{60}$, (E and F) $A_{90}S_{48}O_{46}S_{48}A_{90}$ micelles in dilute aqueous solutions.
